# Leveraging preserved specimens of *Nerodia* to infer the spatiotemporal dynamics of *Ophidiomyces ophidiicola* via quantitative polymerase chain reaction

**DOI:** 10.1002/ece3.9998

**Published:** 2023-04-18

**Authors:** Stephen F. Harding, Maria Del Mar Moretta‐Urdiales, Stephanie C. Nordmeyer, Elijah Wostl, David Rodriguez

**Affiliations:** ^1^ Department of Biology Texas State University San Marcos Texas USA; ^2^ Department of Molecular Immunology and Microbiology University of Texas at San Antonio San Antonio Texas USA; ^3^ Department of Biological Sciences St. Edward's University Austin Texas USA

**Keywords:** EID, museum survey, novel pathogen, qPCR, retrospective, Texas, water snakes

## Abstract

*Ophidiomyces ophidiicola* (*Oo*) is a fungal pathogen and the causative agent of ophidiomycosis that has affected multiple snake taxa across the United States, Europe, and Asia. Ophidiomycosis has often been referred to as an emerging infectious disease (EID); however, its status as an EID has recently come under debate. *Oo* infections have been confirmed in wild snake populations in Texas; however, it is unknown if the pathogen is novel (i.e., invasive) or endemic to the state. To address this knowledge gap, we conducted surveys for *Oo* among preserved *Nerodia* deposited at three university museums in Texas. First, we visually assessed snakes for signs of infection (SOI), and if SOI were present, we sampled the affected area. We then used quantitative polymerase chain reaction to diagnose the presence of *Oo* DNA on areas with SOI and used these data to evaluate spatiotemporal patterns of *Oo* prevalence. We also tested for significant spatial clusters of *Oo* infenction using a Bernoulli probability model as implemented in the program SatScan. We found that the proportion of snakes exhibiting SOI was constant over time while the prevalence of *Oo* DNA among those SOI increased across space and time. Within these data, we detected an incidence pattern consistent with an introduction and then spread. We detected six spatial clusters of *Oo* infection, although only one was significant. Our results support the hypothesis that *Oo* is an emerging, novel pathogen to Texas snakes. These data narrow the knowledge gap regarding the history of *Oo* infections in Texas and establish a historical record of confirmed *Oo* detections in several counties across the state. Thus, our results will guide future research to those areas with evidence of past *Oo* infections but lacking confirmation in contemporary hosts.

## Abstract 2

There is a considerable knowledge gap regarding the spatiotemporal dynamics of *Ophidiomyces ophidiicola* (*Oo*) infection among Texas snakes. To address this knowledge gap, we conducted surveys for signs of *Oo* infection (SOI) among preserved *Nerodia* deposited at three university museums in Texas. We used quantitative polymerase chain reaction to diagnose the presence of *Oo* DNA on snakes with SOI and then used spatiotemporal patterns in these data to evaluate the plausibility of novelty or endemism for *Oo* in this part of North America.

## 1 INTRODUCTION

Emerging infectious diseases (EIDs) are defined as diseases that are novel to science, novel to a population, or are known to be naturally occurring yet suddenly increase in prevalence or range owing to some factor or set of factors (Daszak et al., 2000; Morse, 1995). EIDs caused by fungal pathogens have emerged as threats to plants and animals across the globe (Fisher et al., 2016; Gurr et al., 2011) and are a concern for wildlife because of their potential to trigger the collapse of afflicted populations (Berger et al., 1998; Frick et al., 2010; Lips et al., 2003). Notably, *Batrachochytrium dendrobatidis* (Lips et al., 2006; Vredenburg et al., 2010) and *Batrachochytrium salamandrivorans* (Martel et al., 2013; Spitzen‐van der Sluijs et al., 2013) were linked to amphibian population declines worldwide, while *Pseudogymnoascus destructans* has been linked to the collapse of some North American bat populations (Blehert et al., 2009; Thogmartin et al., 2012).

In snakes, *Ophidiomyces ophidiicola* [formerly *ophiodiicola*] (*Oo*)—the causative fungal pathogen of ophidiomycosis—was first described in 2009 (Rajeev et al., 2009) but may have contributed to the decline of viper populations in New Hampshire in 2006 (Clark et al., 2011) and Illinois in 2008 (Allender et al., 2011). Since then, the pathogen has been detected in wild snakes throughout the Midwest and eastern U.S. (Chandler et al., 2019; Glorioso et al., 2016; Guthrie et al., 2016; Last et al., 2016; Lorch et al., 2016). Recently, *Oo* was detected in Idaho (Allender et al., 2020) and California (Haynes et al., 2021). Thus, it now seems to be distributed across the contiguous U.S. In light of these observations, *Oo* has been referred to as an emerging fungal pathogen of snakes (Allender, Raudabaugh, et al., 2015; Franklinos et al., 2017; Grioni et al., 2021; Guthrie et al., 2016; Lorch et al., 2016; McKenzie et al., 2019; Ohkura et al., 2017). However, a recent study proposed that *Oo* should be viewed as naturally occurring rather than novel, and an unrecognized yet common fungal pathogen of snakes as opposed to a newly emergent pathogen (Davy et al., 2021).

Historically, there are reports of “hibernation blisters” or “hibernation sores” on snakes that emerge from brumation (Clark et al., 2011; Lorch et al., 2016). Other infections may have been responsible for these observations; however, these reports provide anecdotal evidence for the possibility that *Oo* may be naturally occurring or at least has maintained a historical presence in some snake populations. Recently published retrospective surveys for *Oo* in preserved snake specimens corroborated these possibilities by showing evidence that *Oo* infected wild snakes as early as 1945 in the eastern U.S. (Lorch et al., 2021) and 1959 in Europe (Origgi et al., 2022). Using population genetics, Ladner et al. (2022) proposed *Oo* has been introduced to North America multiple times between 1731 and 2012, which could support established historical presence in some populations and potentially, pathogen novelty in others.

In Texas, there is a paucity of data for *Oo*. Presently, six confirmed reports of *Oo* infections were made to Texas Parks and Wildlife (N. Rains & P. Crump, personal communications; see Harding et al., 2022, Dryad repository: https://doi.org/10.5061/dryad.t76hdr83p), one published study showed wide‐spread occurrence among contemporary populations of *Nerodia* in the upper Brazos River drainage (north central Texas) (Harding et al., 2022), and another estimated 15% prevalence among terrestrial and aquatic snakes via SYBR Green qPCR in northeast Texas (Lizarraga et al., 2023). Thus, there is a knowledge gap regarding *Oo* infection dynamics in this region, and it is unknown if *Oo* is naturally occurring and previously unrecognized, naturally occurring but emerging, or has recently spread into Texas (i.e., a novel pathogen).

Evidence supporting these hypotheses could be evaluated by testing for two expected epidemiological patterns. If a pathogen has been introduced and spread, the spatial distribution pattern will be such that there are relatively few cases isolated to only a few areas, then followed by increases in prevalence and distribution across space and time (Cheng et al., 2011; Childs et al., 2000; Guerra et al., 2003; Lips et al., 2008). If a pathogen is naturally occurring, or from a disease ecology perspective—endemic, then evidence of dynamic equilibrium measured as nonchanging pathogen prevalence across space and time would be expected (Becker et al., 2016; Rodriguez et al., 2014).

However, a challenge to addressing historical pathogen dynamics (i.e., support for natural occurrence, endemicity, or novelty) is the availability of samples that retrospectively span several decades. For this purpose, museum collections are advantageous because they provide access to preserved specimens collected at different points in space and time. In the *B. dendrobatidis* system, museum surveys of preserved amphibians have been used to show pathogen emergence concomitant with host population declines in Central America (Cheng et al., 2011); and pathogen endemicity in South America (Becker et al., 2016, Rodriguez et al., 2014). These retrospective studies were useful because they elucidated the contrasting spatiotemporal dynamics of *B. dendrobatidis* infections in Central and South America over several decades and may help explain contemporary patterns in these regions.

Similarly, the goal of our study was to derive support for whether *Oo* is a previously unrecognized, naturally occurring pathogen to Texas snakes, or if it is a recent invader (i.e., novel). To achieve this goal, we inspected preserved snakes for potential SOI and used molecular analyses to determine the presence of *Oo* within this subset. Using these data, we investigated the spatiotemporal dynamics of *Oo* infections in snakes collected across a large part of Texas and tested the plausibility of novelty or natural occurrence for *Oo* in this part of North America.

## 2 MATERIALS AND METHODS

### 2.1 Sample collection

During 2018, 2019, and 2020, we surveyed a total of 2,678 *Nerodia* with “Texas” listed as the state of record from the herpetology collections at the Texas A&M Biodiversity Research and Teaching Collection (TCWC), the University of Texas at Austin Biodiversity Center (TNHC), and the University of Texas at Arlington Amphibian and Reptile Diversity Research Center (ARDRC). Of these specimens, 2,669 were collected across 166 counties. Nine snakes did not have a county of record listed. The sample sizes from TCWC, TNHC, and ARDRC were 891, 873, and 914 snakes, respectively. Taxonomically, we aggregated snakes by species; except *N. harteri*, which was identified to the subspecies level (*N. h. harteri* and *N. h. paucimaculata*). We focused primarily on *Nerodia* spp. for three reasons: (1) the first confirmed report of *Oo* infection in Texas was from *N. h. harteri*, (2) the state contains several widespread species, and (3) to gain historical insight for *Oo* infection associations observed in contemporary *Nerodia* populations by Harding et al. (2022).

Before surveying, the snakes were removed from their jar and placed onto a dissecting tray disinfected with 95% EtOH and wiped clean with fresh paper towels. Then, we visually inspected each snake for potential signs of *Oo* infection (SOI) and assigned a negative (0) if no signs were present or positive (1) if SOI were identified. We defined SOI as the presence of scale abnormalities consistent with signs of ophidiomycosis (e.g., signs of inflammation, dermatitis, gross lesions, crust, or nodules) (Allender, Baker, et al., 2015; Lorch et al., 2015). If a snake showed SOI, it was completely rinsed with fresh 50% EtOH to remove debris. We then sampled affected areas using a single sterile cotton‐tipped swab (Medical Wire, MW113); afterward, we immediately placed the swab into a labeled, sterile screw‐cap tube with an O‐ring. We photographed the dorsum, venter, and lesions for all snakes exhibiting SOI.

To control for false positives, we randomly swabbed one snake with no SOI (i.e., asymptomatic negative controls) for approximately every 10 snakes showing SOI. To assess cross‐contamination between snakes in jars, we selected negative control snakes from jars that also contained snakes showing SOI. Thus, swabs from asymptomatic snakes were collected during the same session(s) and in‐between swabs taken from snakes with SOI. To maximize the area sampled for asymptomatic snakes, we swabbed the entire body starting at the dorsal, anterior end of the snake and then moved towards the posterior using a back‐and‐forth motion. We then repeated this method for the ventral surface. We used a chi‐square test of independence to evaluate the relationship between the presence/absence of SOI and the detection of *Oo*.

### 2.2 DNA extraction, qPCR, and molecular analysis

To extract DNA from the swabs, we used the PrepMan Ultra Sample Preparation Reagent (Applied Biosystems) protocol followed by Harding et al. (2022). Specifically, prior to DNA extraction, the screw‐cap tubes were opened and swabs were allowed to dry for 1 h to ensure that any residual ethanol evaporated. After which, we added 50 μL PrepMan Ultra reagent to each sample, vortexed the tubes for 30 s, and then briefly centrifuged each tube. Then the tubes were boiled at 96–100°C for 10 min and cooled for 2 min. We then centrifuged the samples for 1 min at ≥12,000 *g*. We aseptically inverted the swabs with flame sterilized tweezers in the tubes and centrifuged for another minute at the same velocity to pull the extract out of the swab. The swabs were then aseptically removed and the tubes were centrifuged for 10 min (≥12,000 rcf) to pellet precipitates that might inhibit PCRs. Then, we carefully transferred the supernatant to a new sterile tube leaving behind any precipitates and stored the aliquot at −20°C until processing.

We extracted swabs taken from negative control snakes in the same session as those taken from snakes with SOI. In general, we treated all negative control DNA extracts as experimental samples and included them in the same reaction plate(s) as the DNA extracts from swabs of snakes with SOI. Before conducting qPCR reactions, each sample was diluted 1/10 with nuclease‐free water to reduce the concentration of potential PCR inhibitors. We conducted our reactions utilizing the primers and probe designed by Allender, Bunick, et al. (2015). We used the standards, thermal cycling profile, and reaction protocol described by Harding et al. (2022). We ran all unknown sample reactions in triplicate. We considered a sample positive for *Oo* DNA if the calculated quantity was ≥10 fg (see Harding et al., 2022). Samples that showed no amplification, had calculated quantities <10 fg, or showed aberrant amplification curves were considered negative. We used the Thermo Fisher Connect Cloud Dashboard Software (Thermo Fisher Scientific) for qPCR data processing and analysis.

To control for false negatives caused by low DNA quality owing to the preservation method or other unknown factors, we also conducted end‐point PCRs on the extracts using primers that target a short conserved fragment (~168 bp) within the 16S rRNA gene of Eubacteria (Wang & Qian, 2009), E517F (5′‐GCCAGCAGCCGCGGTAA‐3′) and E685R (5′‐ATCTACGCATTTCACCGCTAC‐3′), with the expectation that bacteria would be common and concomitantly preserved with the specimen. We selected a subset of our samples (*N* = 183) that consisted of snakes with and without SOI collected from 1936 to 2012 and carried out amplifications in 12.5 μL volumes consisting of 0.5 μL of diluted extract (1/10), 6.25 μL of 2X DreamTaq Master Mix (ThermoFisher Scientific), 0.1 mM of each primer, and nuclease‐free water to volume (provided with the DreamTaq kit). Reactions were processed similarly to microbiome PCRs in that they were made in a UV and 10% bleach sterilized biosafety cabinet using dedicated pipettes and barrier tips. All plastic consumables were also UV sterilized prior to use and only unopened reagents were used to minimize the potential for environmental contamination. To test for successful amplification, we electrophoresed the PCR reactions on a 2% agarose gel made with 1X TAE (w/v) in 0.25X TAE buffer at 200 V for 15 min. Reactions were scored based on the presence or absence of the expected band size.

### 2.3 Spatiotemporal analysis

Among the total snakes surveyed, we reported the number that exhibited SOI. For each county and species, we reported the number of *Oo* detections among those with SOI (Table A1 in Appendix 1; Tables 1 and 2). We calculated the proportion of *Oo* detections (i.e., *Oo* prevalence) among all snakes with SOI and for each species surveyed with SOI—and, when applicable, subspecies. We evaluated the strength of detection by estimating the probability of a false‐negative using the equation (1 – *P*)^S^ (Cheng et al., 2011), where *P* was an assumed true *Oo* prevalence value of 5%, 10%, and 20% for a time interval during which *Oo* was not detected and *S* represents the number of qPCR samples (i.e., the number of snakes swabbed).

**TABLE 1:** Texas county of record for preserved *Nerodia* that tested positive for *Ophidiomyces ophidiicola* infection via qPCR, the sample size for each county (*N*), the number of snakes that showed signs of infection (SOI), the number that tested positive of *O. ophidiicola* DNA (*Oo* +), and the earliest year an *Oo* + snake was captured.

County	*N*	SOI	*Oo* +	Earliest detection
Bowie	4	1	1	2014
Brazoria	57	4	1	1962
Brown	4	1	1	1993
Calhoun	8	1	1	1999
Coke	126	30	4	1960
Collin	7	1	1	–
Concho	98	20	10	1961
Dallas	74	16	6	1998
Fannin	11	1	1	1993
Fisher	1	1	1	2009
Franklin	4	1	1	2000
Galveston	94	19	2	1955
Hardin	21	4	1	1979
Hill	11	1	1	2007
Houston	12	1	1	1993
Jasper	14	2	1	1972
Johnson	14	2	2	1979
Kaufman	7	1	1	–
Marion	5	1	1	1984
McLennan	18	5	1	–
Menard	29	2	1	2008
Newton	7	2	1	1986
Palo Pinto	106	26	2	1987
Parker	29	6	6	1989
Rains	5	1	1	1996
Runnels	160	13	7	1961
Sabine	4	1	1	1992
San Saba	20	4	1	2017
Smith	51	7	2	1986
Somervell	23	1	1	1987
Tarrant	89	6	2	2012
Titus	24	1	1	1993
Trinity	37	3	2	1988
Tyler	12	1	1	1993

*Note*: The year of earliest detection is provided for each county unless the specimen did not have a date of collection.

**TABLE 2:** Species summary of the number of preserved *Nerodia* (*N*) surveyed for signs of infection (SOI), the prevalence of SOI (SOI/*N*), 95% binomial confidence intervals (SOI CI), the number of *Oo* + snakes, *Oo* prevalence among those with SOI (*Oo* +/SOI), and 95% binomial confidence intervals (*Oo* + CI).

Taxon	*N*	SOI	SOI/*N*	SOI CI	*Oo* +	*Oo*+/SOI	*Oo* + CI
*N. clarkii*	299	25	0.08	0.06–0.12	2	0.08	0.02–0.27
*N. cyclopion*	92	8	0.09	0.04–0.16	1	0.13	0.02–0.54
*N. erythrogaster*	936	81	0.09	0.07–0.11	16	0.20	0.13–0.30
*N. fasciata*	292	30	0.10	0.07–0.14	7	0.23	0.12–0.42
*N. harteri harteri*	113	37	0.33	0.25–0.42	8	0.22	0.11–0.38
*N. harteri paucimaculata*	394	66	0.17	0.13–0.21	21	0.32	0.22–0.44
*N. rhombifer*	544	50	0.09	0.07–0.12	8	0.16	0.08–0.29
*N. sipedon*	8	5	0.63	0.28–0.87	5	1.00	0.48–1.00

To visualize temporal patterns of SOI and *Oo* prevalence and spatiotemporal patterns of *Oo* detections, we aggregated snakes by the year collected in the following manner: 1905–1954, 1955–1959, and then at 10‐year intervals afterward. We tested for significant *Oo* infection clusters across space using a Bernoulli probability model implemented in SaTScan v9.6 (Kulldorff & Nagarwalla, 1995). Because several snakes (*N* = 99) did not have a date of collection associated with their catalog number and the sampling distribution was not even across time, we did not conduct a temporal cluster analysis. We defined the maximum spatial cluster size as 50% of the population with a maximum radius of 50 km. To maximize the sample size for each county, we aggregated data points into time intervals of 10 years.

We visualized the data using R software (R Core Team, 2021). We constructed data frames using the “readxl” (Wickham & Bryan, 2019), “plyr” (Wickham, 2011), “dplyr” (Wickham et al., 2019), and “reshape” (Wickham, 2007) R packages. We estimated the 95% binomial confidence intervals with a logistic parameterization for grouping data (i.e., species) using the R package “binom” (Dorai‐Raj, 2014). Maps were constructed using the R packages: “ggplot2” (Wickham, 2016), “ggmap” (Kahle & Wickham, 2013), “maps” (Becker, Wilks, Brownrigg, et al., 2018), “mapdata” (Becker, Wilks, & Brownrigg, 2018), “maptools” (Bivand & Lewin‐Koh, 2019), “sf” (Pebesma, 2018), “tmap” (Tennekes, 2018), and “tmaptools” (Tennekes, 2019).

## 3 RESULTS

**FIGURE 1:**
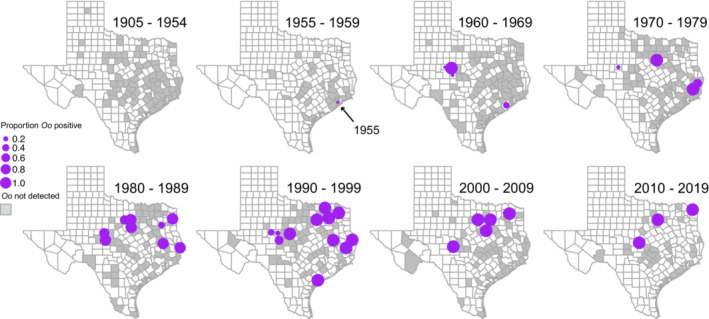
Distribution of the proportion of *Ophidiomyces ophidiicola* detections (*Oo* +) among preserved *Nerodia* with potential signs of *Oo* infection (SOI) collected in Texas from 1905 through 2019. The size of the closed circle is scaled to the proportion of *O. ophidiicola* detections for each county. The county of record (shaded gray) for preserved snakes when SOI or *O. ophidiicola* were not observed or detected. The earliest detection (1955) is indicated by the arrow.

**FIGURE 2:**
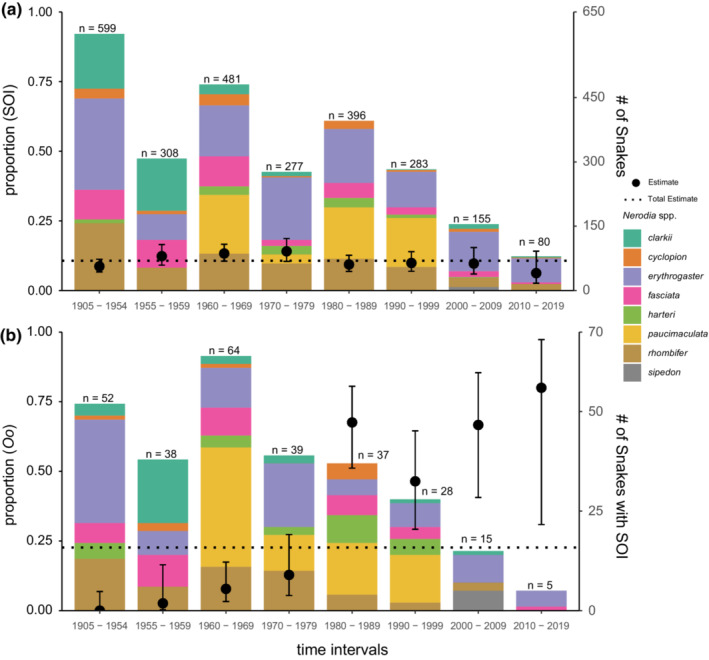
Preserved Texas *Nerodia* species collected from 1905 through 2019 grouped by time intervals and surveyed for signs of *Ophidiomyces ophidiicola* infection (SOI). (a) The proportion of *Nerodia* that exhibited SOI; and (b) the prevalence of *O. ophidiicola* among those snakes exhibiting SOI. The prevalence of SOI and *O. ophidiicola* prevalence for all *Nerodia* surveyed are indicated by the dotted line.

Of the snakes we surveyed (*N* = 2678), SOI were present on 302 (11.3%; CI: 10.1%–12.5%). Among those with SOI, 68 tested positive for the presence of *Oo* (22.3%; CI: 18.5%–27.6%), these were collected from 34 counties (Table 1). We did not detect *Oo* on snakes collected from 1905 to 1954 (Figures 1 and 2b) and the earliest *Oo* detection was from a snake collected in Galveston Co. in 1955 (Table 1). We successfully amplified bacterial DNA for 181 of 183 samples collected from 1936 to 2012 and the negative control reaction showed no amplification (see Supporting Data).

We detected a significant relationship between the presence/absence of SOI and the detection of *Oo, X* ^*2*^ (1, *N* = 398) = 5.77, *p* < .01. Of the 26 asymptomatic negative control swabs, none of them tested positive for the presence of *Oo*. Thus, they were omitted from downstream analyses of *Oo* prevalence. Therefore, our *Oo* prevalence estimates for *Nerodia* collected in Texas were based on 302 snakes showing SOI (Table 2). *Oo* prevalence for *Nerodia* spp. ranged from 8.0% (*N. clarkii*) to 100% (*N. sipedon*) (Table 2). Species‐level *Oo* prevalence for *N. sipedon* was significantly different from mean *Oo* prevalence while *Oo* prevalence for *N. h. paucimaculata* approached significance (Table 2). All species surveyed had at least one positive detection (Table 2).

**FIGURE 3:**
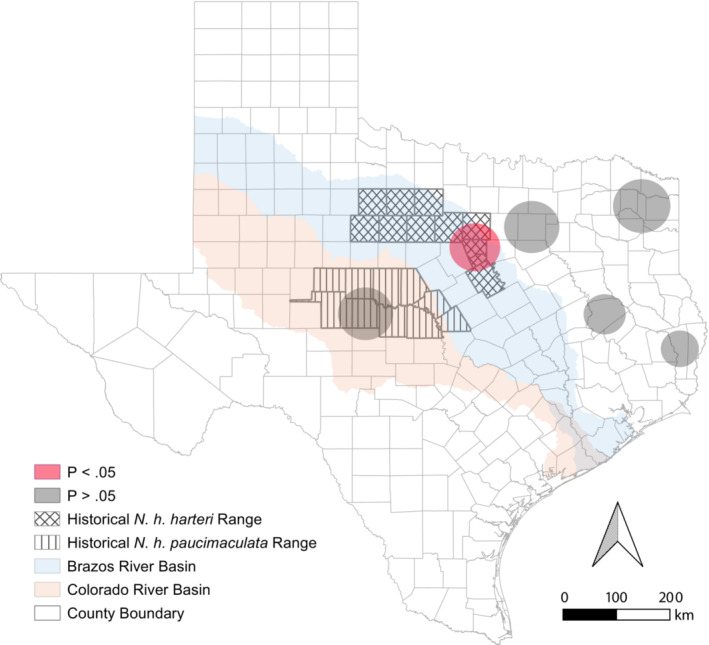
Mapped clusters (red = statistically significant; gray = not significant) derived from SaTScan spatial analysis of *Ophidiomyces ophidiicola* detections among preserved Texas *Nerodia*. Hatching demarcates the counties encompassing the range of *N. harteri*.

The proportion of *Oo* detections among preserved Texas *Nerodia* with SOI and the spatial distribution of those detections increased from 1955 through 2019 (Figure 1). The proportion of snakes that showed SOI was constant across time (Figure 2a). Relative to all time periods from 1905–1979, *Oo* prevalence increased between 1980–1989, 1990–1999, 2000–2009, and 2010–2019 (Figure 2b). Our estimated probability of a false‐negative (i.e., failure to detect *Oo* when it is present) for samples collected between 1905 and 1954 with an assumed true prevalence of 5%, 10%, and 20% was <0.001% for all three assumptions. Spatial analysis of *Oo* prevalence resulted in six clusters; however, only one was significant (Figure 3).

## 4 DISCUSSION

By surveying preserved *Nerodia* spp. from three museum collections, we have shown that *Oo* has been widespread and infecting snakes in Texas since at least 1955—approximately 53 years before first being detected in wild snake populations in the eastern U.S. (Allender et al., 2011; Rajeev et al., 2009). In general, *Oo* prevalence among our samples increased (Figures 1 and 2b) over time, while the prevalence of SOI was consistent (~11%) (Figure 2a). The spatiotemporal patterns for *Oo* prevalence in our study are consistent with a pattern of introduction—or multiple introductions—and spread rather than a pattern indicative of long‐term presence (i.e., endemism or natural occurrence). We also detected evidence of past outbreaks within *Nerodia harteri*, a Texas endemic species of conservation concern.

### 4.1 Historical pathogen dynamics

We might expect that SOI would also increase temporally if *Oo* was an invasive pathogen; however, we observed a stable prevalence of SOI over space and time. This pattern could have been due to sampling bias when these snakes were initially collected, or it could have also resulted from our inclusive approach to surveying for the presence of *Oo*. During the early stages, *Oo* infection is sometimes indicated by mild dermatitis or a subtle crust. Therefore, we did not constrain our methods to target snakes with specific lesion types or the severity or number of lesions. Consequently, it is possible that we identified and sampled snakes with skin wounds or lesions not infected with or caused by *Oo*. If *Oo* invaded this region, prior disruption of the dermal layer via naturally occurring injuries may have served as opportunistic pathways for infection given that experimental infections are sometimes initiated via dermal abrasion (Lorch et al., 2015). In this case, SOI, as we broadly defined it, would remain relatively constant, yet prevalence of *Oo* would increase.

Nonetheless, our observations are consistent with other retrospective studies that have shown *Oo* infections have been present in wild snakes in the eastern U.S. since 1945 (Lorch et al., 2021) and in Europe since 1959 (Origgi et al., 2022). Thus, ophidiomycosis has indeed gone unrecognized or overlooked in Texas and other regions for decades. However, our results showed *Oo* prevalence among our samples increased significantly starting in 1980 (Figure 2), and snakes across more counties were infected when compared to the period from 1905 through 1979 (Figure 1). This spatiotemporal pattern of increasing prevalence is consistent with epizootic outbreaks shown in studies of *B. dendrobatidis* (Brem & Lips, 2008; Cheng et al., 2011; Lips et al., 2006) and contrasts with studies showing enzootic patterns of pathogen prevalence (Becker et al., 2016; Rodriguez et al., 2014).

Ladner et al. (2022) hypothesized that there may have been multiple recent introductions of *Oo* in North America. Specifically, they proposed that one lineage of *Oo* was introduced into North America sometime between 1731 and 2012, while two others were introduced between 1902 and 2009. Lorch et al. (2021) and this study support the conclusions of Ladner et al. (2022). Another study comparing the genetic similarity between *Oo* isolates collected in Texas, the eastern U.S., and Europe showed evidence of shared genotypes between Texas, Massachusetts, Maryland, and New York (Harding, 2022). Collectively, these results are consistent with the hypothesis that *Oo* spread across North America, possibly via human‐mediated transport, and that *Oo* is a recently introduced pathogen in Texas.

### 4.2 Detection of *Oo*

The absence of *Oo* detections on snakes collected across Texas from 1905 to 1954 (Figure 1) could be, in part, false negatives rather than an absence of *Oo*. Two factors that can contribute to false negatives are DNA degradation and failing to capture *Oo* during sampling. Regarding DNA degradation, we successfully and reliably amplified bacterial 16S rDNA from preserved snakes. This indicated that our methods extracted DNA sufficient for molecular analyses even from non‐target organisms. Additionally, other retrospective surveys that used skin swabbing methods similar to ours sequenced a DNA fragment of approximately 146 bp from *B. dendrobatidis* amplified from preserved specimens collected in 1863 (Burrowes & De la Riva, 2017) and 1894 (Rodriguez et al., 2014), which were decades older than the earliest specimen in our study. Therefore, we reasonably assume DNA degradation was not a significant factor when accounting for potential false negatives in our study.

Regarding false negatives owing to a failed extraction of *Oo* DNA, when we assumed a true prevalence (20%) comparable to our observed estimated prevalence (~22%), our estimated probability of a false negative for our sample size was low (<0.001%). Comparatively, our methods reported here were based on our *Oo* sampling methods for live snakes where our estimated false negative rate was ~15% (Harding et al., 2022). Explicitly, we surveyed 599 snakes collected in Texas from 1905 to 1954, of which 52 showed SOI. Considering the probability of false negatives in these results and our aforementioned false‐negative rate, we estimated up to 8 samples from this period are true positives but tested negative. Thus, we assume that SOI observed on snakes collected before 1955 were caused by something other than *Oo*, and our failure to detect *Oo* DNA is more likely owing to the absence of *Oo* infections rather than false negatives.

### 4.3 Conservation implications

Subspecies of *N. harteri* are taxa of conservation concern characterized by restricted ranges, low abundance, and low genetic diversity (Janecka et al., 2021; Rodriguez et al., 2012). Our retrospective estimates of *Oo* prevalence for *N. h. paucimaculata* (32.0%, CI: 22.0%–44.0%) and *N. h. harteri* (22.0%, CI: 11.0%–38.0%) were moderately high (Table 2). Additionally, infection clusters could represent past ophidiomycosis outbreaks, and our spatial scan analysis detected a significant cluster (*p* < .05) within the range of *N. h. harteri* (Hood, Parker, and Somervell counties), and a non‐significant (*p* > .05) cluster within the range of *N. h. paucimaculata* (Concho and Runnels counties) (Figure 3). These results are notable because populations of *N. harteri* have been historically affected by multiple anthropogenic and natural stressors (McBride, 2009; Scott et al., 1989), which may have contributed to decreased abundance (McBride, 2009; Rodriguez et al., 2012). In addition to these population stressors, we have provided evidence of overlooked pathogenic stressors in *N. harteri* and now propose that ophidiomycosis may have also contributed to low abundance in these populations. This is significant because our retrospective study corroborates high contemporary *Oo* infection estimates for adult *N. h. harteri* (94.4%) and should be considered in future management decisions for this species (Harding et al., 2022).

Our *Oo* prevalence estimates for *Nerodia* likely do not reflect the true prevalence of *Oo* infections in Texas snakes across either space or time. Indeed, wide confidence intervals reflect the uncertainty in some of our estimates (Figure 2b; Table 2). However, our study narrows the knowledge gap regarding the history of *Oo* infections in Texas *Nerodia* and provides additional insight into contemporary *Oo* host‐pathogen dynamics observed in *N. harteri* populations. Even though we have addressed historical *Oo* infection dynamics for *Nerodia*, ophidiomycosis remains understudied for most of the snake species and areas in Texas. Therefore, retrospective and contemporary surveys at the population level that include other snake taxa are still needed. We have begun to characterize the spatiotemporal distribution of *Oo* infections in this region, which will guide future research to those areas with evidence of past *Oo* infection but lacking confirmation of contemporary infections.

## AUTHOR CONTRIBUTIONS

**Stephen Forrest Harding:** Conceptualization (equal); data curation (equal); formal analysis (equal); methodology (equal); supervision (supporting); validation (equal); visualization (equal); writing – original draft (equal); writing – review and editing (equal). **Maria Del Mar Moretta‐Urdiales:** Formal analysis (equal); methodology (equal); writing – original draft (equal); writing – review and editing (equal). **Stephanie Nordmeyer:** Data curation (equal); writing – review and editing (equal). **Elijah Wostl:** Data curation (equal); writing – review and editing (equal). **David Rodriguez:** Conceptualization (equal); data curation (equal); funding acquisition (lead); methodology (equal); resources (equal); supervision (lead); validation (equal); visualization (equal); writing – original draft (equal); writing – review and editing (equal).

## FUNDING INFORMATION

This project was funded by startup funds to D.R. from Texas State University.

## CONFLICT OF INTEREST STATEMENT

All authors have no competing interests to declare.

## Data Availability

Supporting data and additional photographs documenting signs of infection on specimens are available at our Zenodo repository: 10.5281/zenodo.7775088.

## APPENDIX 1

**TABLE A1:** Texas county of record for preserved *Nerodia* surveyed for signs of *Ophidiomyces ophidiicola* infection (SOI).

County	*N*	SOI	*Oo* +	Earliest detection
Anderson	15	4	–	–
Angelina	3	–	–	–
Aransas	15	1	–	–
Archer	4	–	–	–
Austin	6	1	–	–
Bandera	8	–	–	–
Bastrop	12	2	–	–
Baylor	2	–	–	–
Bee	4	–	–	–
Bell	8	–	–	–
Bexar	7	1	–	–
Blanco	6	–	–	–
Bosque	3	–	–	–
Bowie	4	1	1	2014
Brazoria	57	4	1	1962
Brazos	42	1	–	–
Brewster	1	–	–	–
Brown	4	1	1	1993
Burleson	63	2	–	–
Burnet	4	–	–	–
Caldwell	3	1	–	–
Calhoun	8	1	1	1999
Callahan	1	–	–	–
Cameron	8	–	–	–
Camp	1	–	–	–
Cass	2	–	–	–
Chambers	104	6	–	–
Childress	1	–	–	–
Clay	3	1	–	–
Coke	126	30	4	1960
Coleman	34	8	–	–
Collin	7	1	1	NA
Colorado	37	–	–	–
Comal	10	3	–	–
Comanche	3	–	–	–
Concho	98	20	10	1961
Cooke	3	–	–	–
Coryell	1	–	–	–
Cottle	1	–	–	–
Dallas	74	16	6	1998
Dawson	8	1	–	–
De Witt	5	–	–	–
Delta	4	–	–	–
Denton	23	1	–	–
Eastland	3	–	–	–
Eastman	1	–	–	–
Edwards	1	–	–	–
Ellis	1	–	–	–
Erath	3	–	–	–
Fannin	11	1	1	1993
Fayette	2	–	–	–
Fisher	1	1	1	2009
Foard	1	–	–	–
Fort Bend	8	–	–	–
Franklin	4	1	1	2000
Freestone	29	–	–	–
Frio	1	1	–	–
Galveston	94	19	2	1955
Garza	1	–	–	–
Gillespie	1	–	–	–
Goliad	1	–	–	–
Gonzales	25	4	–	–
Grayson	6	1	–	–
Gregg	1	–	–	–
Grimes	5	–	–	–
Guadalupe	2	–	–	–
Hamilton	1	–	–	–
Hardin	21	4	1	1979
Harris	252	12	–	–
Harrison	8	–	–	–
Haskell	1	–	–	–
Hays	18	2	–	–
Henderson	8	–	–	–
Hill	11	1	1	2007
Hood	8	2	–	–
Hopkins	5	–	–	–
Houston	12	1	1	1993
Hunt	3	–	–	–
Hutchinson	1	–	–	–
Jack	3	–	–	–
Jackson	2	1	–	–
Jasper	14	2	1	1972
Jefferson	52	4	–	–
Johnson	14	2	2	1979
Jones	2	–	–	–
Karnes	1	–	–	–
Kaufman	7	1	1	NA
Kendall	1	–	–	–
Kerr	7	–	–	–
Kimble	4	1	–	–
La Salle	3	–	–	–
Lamar	2	–	–	–
Lampasas	4	–	–	–
Lavaca	3	–	–	–
Lee	2	–	–	–
Leon	22	2	–	–
Liberty	12	2	–	–
Live Oak	2	–	–	–
Llano	23	–	–	–
Lubbock	3	–	–	–
Madison	5	1	–	–
Marion	5	1	1	1984
Mason	11	4	–	–
Matagorda	9	3	–	–
Maverick	1	–	–	–
McCulloch	17	–	–	–
McLennan	18	5	1	NA
McMullen	2	1	–	–
Medina	1	–	–	–
Menard	29	2	1	2008
Milam	5	2	–	–
Mitchell	1	–	–	–
Montague	1	–	–	–
Montgomery	19	–	–	–
Morris	1	–	–	–
Nacogdoches	6	–	–	–
Navarro	15	2	–	–
Newton	7	2	1	1986
Nueces	2	–	–	–
Orange	27	4	–	–
Palo Pinto	106	26	2	1987
Panola	1	–	–	–
Parker	29	6	6	1989
Polk	5	1	–	–
Rains	5	1	1	1996
Real	1	–	–	–
Red River	5	–	–	–
Reeves	4	1	–	–
Refugio	4	–	–	–
Robertson	6	–	–	–
Rockwall	2	–	–	–
Runnels	160	13	7	1961
Rusk	1	1	–	–
Sabine	4	1	1	1992
San Jacinto	10	–	–	–
San Patricio	6	–	–	–
San Saba	20	4	1	2017
Shackelford	6	–	–	–
Shelby	2	–	–	–
Smith	51	7	2	1986
Somervell	23	1	1	1987
Starr	1	–	–	–
Stephens	6	–	–	–
Tarrant	89	6	2	2012
Taylor	1	–	–	–
Terrell	13	–	–	–
Throckmorton	4	1	–	–
Titus	24	1	1	1993
Tom Green	3	–	–	–
Travis	108	13	–	–
Trinity	37	3	2	1988
Tyler	12	1	1	1993
Uvalde	3	–	–	–
Val Verde	3	–	–	–
Victoria	3	–	–	–
Walker	116	9	–	–
Waller	4	1	–	–
Washington	11	1	–	–
Webb	1	–	–	–
Wharton	4	1	–	–
Wichita	2	–	–	–
Williamson	12	–	–	–
Wise	6	–	–	–
Wood	4	–	–	–
Zapata	2	–	–	–
Zavala	2	–	–	–

*Note*: Listed for each locality are the number of snakes surveyed (*N*), the number of snakes with SOI, the number of snakes with SOI that tested positive of *O. ophidiicola* DNA (*Oo* +), and the earliest year in which a snake was collected that exhibited SOI and tested positive for *O. ophidiicola* DNA.
